# A new scoring system for predicting extent of resection in medial sphenoid wing meningiomas based on three-dimensional multimodality fusion imaging

**DOI:** 10.1186/s41016-020-00214-0

**Published:** 2020-11-02

**Authors:** Zilan Wang, Xiaolong Liang, Yanbo Yang, Bixi Gao, Ling Wang, Wanchun You, Zhouqing Chen, Zhong Wang

**Affiliations:** 1 Department of Neurosurgery, The First Affiliated Hospital of Soochow University, 188 Shizi Street, Suzhou, 215006 Jiangsu Province China; 2 Department of Orthopedics, The First Affiliated Hospital of Soochow University, Suzhou, 215006 Jiangsu Province China; 3 Department of Radiology, Suzhou Hospital of Traditional Chinese Medicine, Suzhou, 215006 Jiangsu Province China

**Keywords:** Brain tumor, Sphenoid wing meningioma, Scoring system, Fusion imaging, 3D reconstruction

## Abstract

**Background:**

Three-dimensional (3D) fusion imaging has been proved to be a promising neurosurgical tool for presurgical evaluation of tumor removal. We aim to develop a scoring system based on this new tool to predict the resection grade of medial sphenoid wing meningiomas (mSWM) intuitively.

**Methods:**

We included 46 patients treated for mSWM from 2014 to 2019 to evaluate their tumors’ location, volume, cavernous sinus involvement, vascular encasement, and bone invasion by 3D multimodality fusion imaging. A scoring system based on the significant parameters detected by statistical analysis was created and evaluated.

**Results:**

The tumor volumes ranged from 0.8 cm^3^ to 171.9 cm^3^. A total of 39 (84.8%) patients had arterial involvement. Cavernous sinus (CS) involvement was observed in 23 patients (50.0%) and bone invasion was noted in 10 patients (21.7%). Simpson I resection was achieved in 10 patients (21.7%) and Simpson II resection was achieved in 17 patients (37.0%). Fifteen patients (32.6%) underwent Simpson III resection and 4 patients (8.7%) underwent Simpson IV resections. A scoring system was created. The score ranged from 1 to 10 and the mean score of our patients was 5.3 ± 2.8. Strong positive monotonic correlation existed between the score and resection grade (*R*_s_ = 0.772, *P* < 0.001). The scoring system had good predictive capacity with an accuracy of 69.60%.

**Conclusions:**

We described a scoring system that enabled neurosurgeons to predict extent of resection and outcomes for mSWM preoperatively with 3D multimodality fusion imaging.

**Trial registration:**

Retrospectively registered

## PACS picture archiving and communication system

SD Standard deviation

SWM Sphenoid wing meningiomas

3D Three-dimensional

## Background

Sphenoid wing meningiomas (SWM) make up approximately 10–15% of total cranial meningiomas [14]. Medial sphenoid wing meningiomas (mSWM) present a surgical challenge because they can grow into the cavernous sinus (CS), encircle the anterior circulation arteries, affect the cranial nerves, and even invade the bone [16, 25]. The intricate location of medial sphenoid wing meningiomas (mSWM) increases the risk of surgery, leading to higher morbidity and even mortality. It is crucial to study preoperative imaging to predict which extent of resection can be achieved and decide whether to manage total resection for lower recurrence rate or partial resection for preservation of encased neurovascular structures. We have not yet had a widely accepted classification system of mSWM to predict the extent of resection in clinical practice. Recently, application of three-dimensional (3D) multimodality fusion imaging has greatly contributed to the understanding of anatomical structures and has been proved to be a promising neurosurgical tool for brain tumors [7, 17, 21]. So, our objective is to develop a scoring system based on the preoperative 3D multimodality fusion imaging to predict the grade of tumor resection.

## Methods

### Patients and study design

The inclusion criteria for the study were the presence of medial sphenoid wing meningiomas based on radiologic, intraoperative, and pathologic findings from 2014 to 2019. Meningiomas originating in the tuberculum sellae, orbital roof, cavernous sinus, or middle or lateral aspects of the sphenoid wing were excluded. Each patient included needed a preoperative computed tomography (CT), magnetic resonance imaging (MRI) with and without contrast, and magnetic resonance angiography (MRA)/computed tomography angiography (CTA). The study was approved by the local ethics committee as a retrospective study (Institutional Review Board No. 2019117). As this article is a retrospective study, ethics committees have been granted exemption of patient informed consent.

### Image processing

The Digital Imaging and Communications in Medicine (DICOM) data format of images was imported from our institution’s Picture Archiving and Communication System (PACS) (Neusoft Corp., Shenyang, China) into Mimics Medical 15.0 (Materialise Company, Leuven, Belgium). Firstly, we created a mask of the imaging data to extract the targeted structure by determining the region of interest and adjusting the thresholding. Skull was delineated from CT. Blood vessels were delineated from MRA/CTA. Tumor was delineated from the T1-weighted of MRI with contrast. Then, edits were done on the masks and the unclear margins were outlined manually. Finally, all the targeted structures were integrated into the same space and manually adjusted to the exact place to generate the 3D multimodality fusion imaging data. Tumors were set as transparent to assess the arteries inside. Tumor location, volume, CS involvement, vascular encasement, and bone invasion were evaluated. All the images were evaluated by a neuroradiologist and a young neurosurgeon, without knowledge of any patients’ clinical data.

**Fig. 1:**
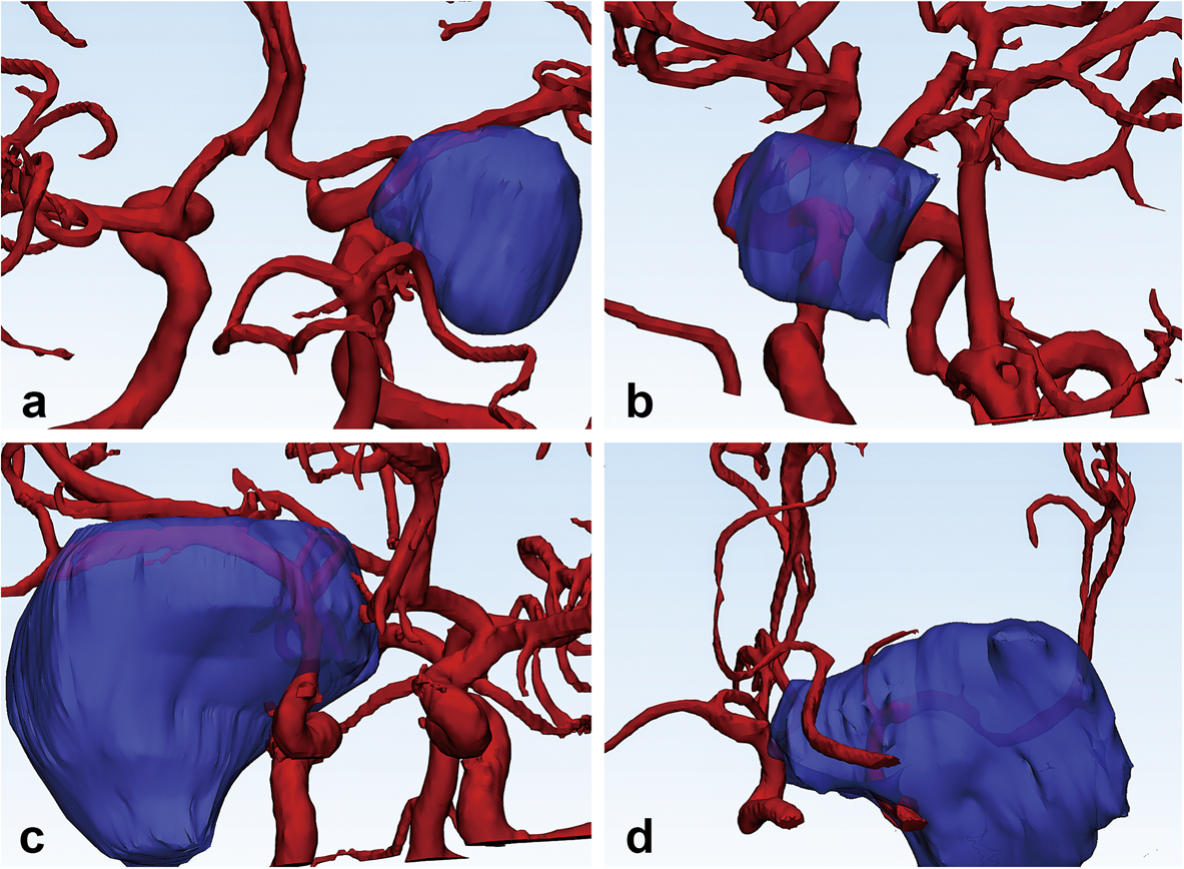
Three-dimensional multimodality medical fusion imaging demonstrating the arterial involvement and cavernous sinus involvement. **a** The tumor partially encircling the M1 segment of the middle cerebral artery (MCA) without narrowing the lumen, with a score of 1. **b** The tumor completely (360°) encircling the internal carotid artery (ICA) without narrowing the lumen, with a score of 2. **c** The tumor completely encircling and narrowing the M1 segment of the MCA, with a score of 3. **d** The tumor completely encircling and narrowing the ICA and M1 segment of the MCA, with a score of 4

**Fig. 2:**
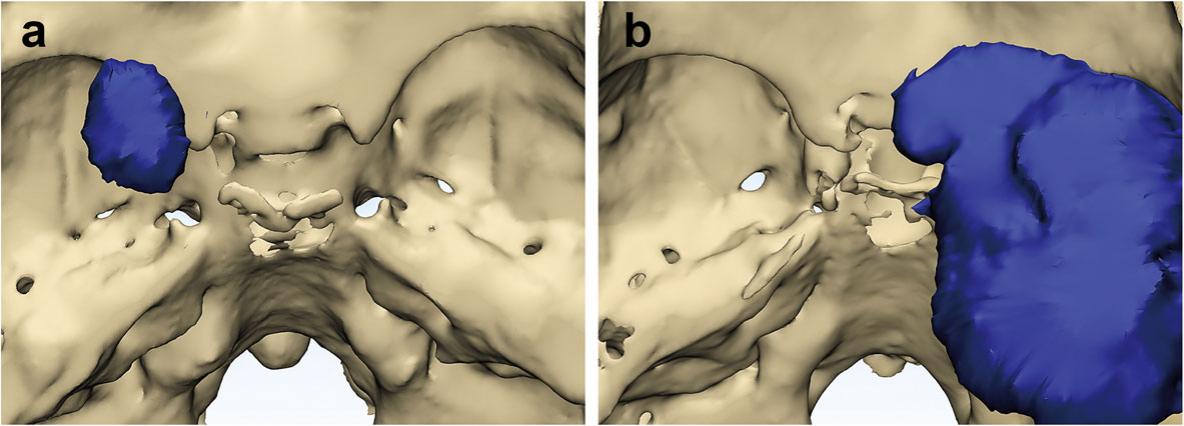
**a** Tumor without cavernous sinus involvement. **b** Tumor with cavernous sinus involvement

*The volume of the tumor* was calculated after we established the 3D model of the tumor by Mimics software. Volume < 20 cm^3^ was categorized into grade 1, volume between 20 and 50 cm^3^ was categorized into grade 2, and volume > 50 cm^3^ was categorized into grade 3. Grade of *arterial encasement* was determined as follows: involvement of any artery was given one point, and an additional point was given for each of the following features as shown in Fig. 1: completely encircling (360°) the artery, involving more than one artery, and narrowing the lumen of the artery. The grade ranged from 0 to 4. *CS involvement* was defined as infiltration of the tumor into the cavernous sinus as shown in Fig. 2. *Bone invasion* was observed by the 3D reconstruction of the skull.

### Microsurgical technique

Three neurosurgeons performed these surgeries and each neurosurgeon performed 27 (58.7%), 11 (23.9%), and 8 (17.4%) cases, respectively. For microsurgical tumor removal, pterional approach was performed on 45 patients (98%) and lateral supraorbital approach was performed on 1 patient (2%). A high-speed pneumatic drill was used to remove any thickened bony prominence of the lesser and greater sphenoid wings. After opening the dura, the operating microscope was used, and the tumor was approached through the sylvian fissure. The basal dural attachment of the tumor on the sphenoid ridge was coagulated to decrease the tumor’s blood supply and detach it from the dura. The capsule of the tumor was then opened, and the tumor was resected piecemeal. After resection of the tumor, the dura attachment was coagulated.

### Extent of tumor resection

The extent of tumor resection was categorized according to the Simpson grade of resection [23]: grade I, total tumor resection with excision of its dural attachment, and any abnormal bone; grade II, total tumor resection and coagulation of dural attachments; grade III, gross total tumor resection without resection or coagulation of its dural attachment, or alternatively, of its extradural extensions, e.g., an invaded sinus or hyperostotic bone; grade IV, a partial removal, leaving intradural tumor in situ; and grade V, a simple decompression. The Simpson grade of our cases was determined by the surgical records, videos, and postoperative imaging studies.

### Postoperative symptoms and follow-up

All the symptoms observed after surgery were recorded. To evaluate the outcome of surgical treatment, patients were assigned to a 4-category postoperative evaluation system used by Honig et al [11]: grade 0 represents a worse outcome with deterioration of symptoms, grade 1 is a fair outcome with unchanged symptoms, and grade 2 is a good outcome with improvement of symptoms. Patients with grade 3 show an excellent outcome with complete regression of preoperative tumor-related symptoms.Statistical analyses

The variables included the volumes of the tumor, CS involvement, arterial involvement, and bone invasion. Kruskal-Wallis test for ordinal variables such as tumor volume grade and Pearson’s chi-square test for nominal variables such as bone invasion were performed to compare between four different resection grade groups. Descriptive continuous data were reported as mean ± standard deviation (SD), and categorical data were reported as percentages. The adjusted odds ratios (OR) were obtained by a logistic regression model including the variables showing significant differences in univariate analysis (*P* < 0.05). The correlation between the variables and resection grade was detected respectively by Spearman’s correlation analysis. The predictive capacity of our scoring system was evaluated by discriminant analysis. The results were evaluated at 95% confidence interval (95% CI) and *P* < 0.05 was considered statistically significant. Statistical analyses were performed using SPSS version 25.0 software (IBM Corp., Armonk, New York, USA).

## Results

### Study population and preoperative findings

A total of 1316 meningiomas were operated between 2014 and 2019 and 168 cases were treated for the diagnosis of SWM. In total, 91 patients were identified and received surgeries for mSWM, but only 46 patients were included in this study. The other patients were excluded from the study for lack of preoperative radiologic imaging availability in our PACS system for review. Among the 46 patients, 31 were women (67.4%) and 15 were men (32.6%). The patients’ ages ranged from 27 to 80 years (mean, 56.5 ± 12.2 years). The most frequent symptom was visual deterioration (*n* = 29, 63.0%) followed by headache (*n* = 14, 30.4%) and dizziness (*n* = 10, 21.7%). A summary of the patients’ clinical characteristics was presented in Table 1. Four patients received an operation before 2014 and underwent reoperation during the past 5 years because of regrowth of meningioma.

**Table 1:** Clinical data from 46 patients with medial sphenoid wing meningiomas

Clinical features	*N* (%)
Female	31 (67.4)
Male	15 (32.6)
Age, mean ± SD (year) (range)	56.5 ± 12.2 (27-80)
Symptoms^a^	
Ataxy	5 (10.9)
Dizziness	10 (21.7)
Facial numbness	2 (4.3)
Headache	14 (30.4)
Memory impairment	8 (17.4)
Motor weakness	9 (19.6)
Ptosis	4 (8.7)
Proptosis	2 (4.3)
Seizures	3 (6.5)
Visual field defect	6 (13.0)
Visual deterioration	29 (63.0)

^a^Some patients had multiple symptoms

*SD* standard deviation

### Radiologic findings

**Fig. 3:**
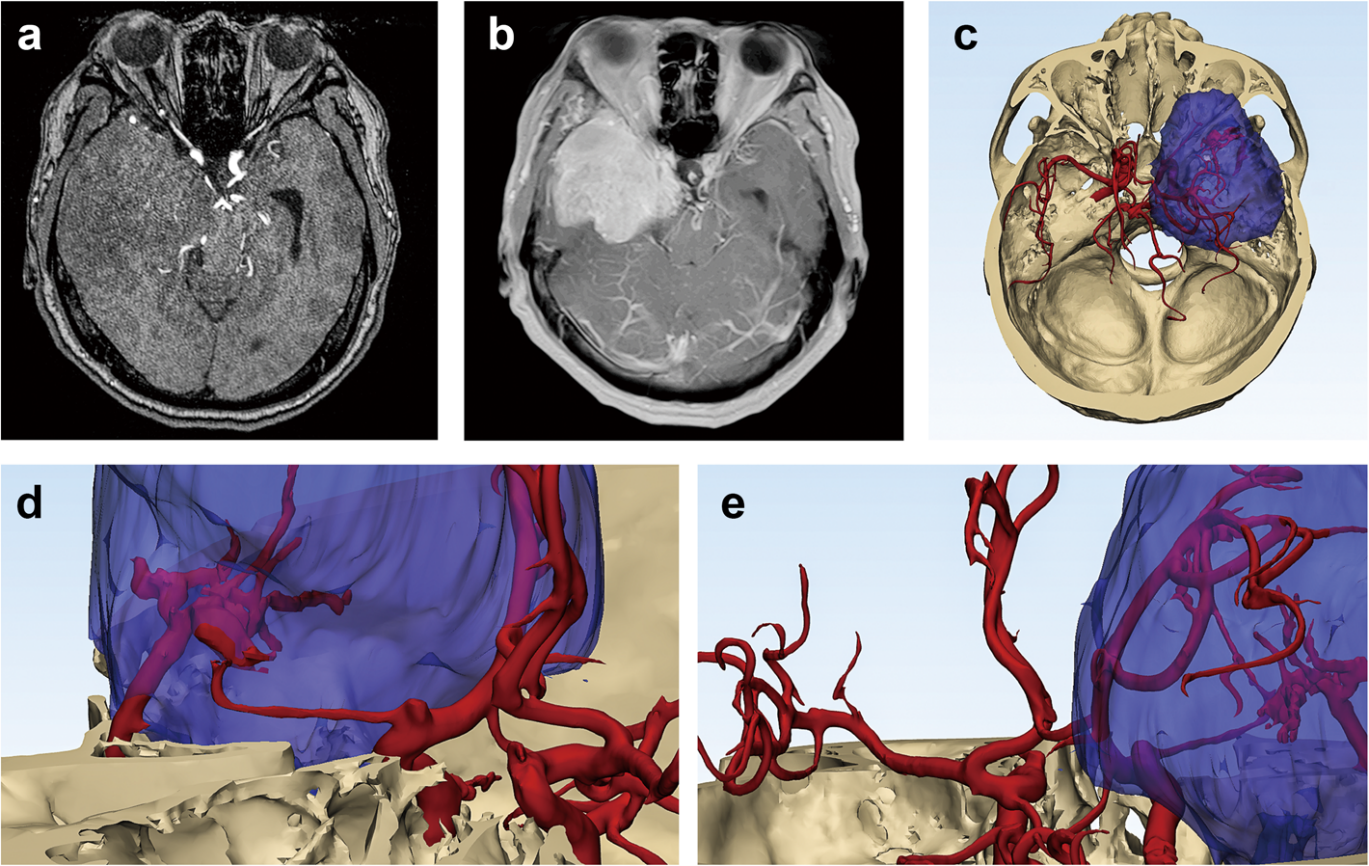
A patient who complained about the weakness of lower extremities for half a year. **a** Axial view on MRA showing the C4 segment of ICA and M1 segment of MCA displaced by the tumor. **b** Axial view on MRI showing a giant right medial sphenoid wing meningioma. **c** The 3D structures of brain tumor (purple), arteries (red), and bone structure (beige) reconstructed and imaged by Mimics software showing the tumor grew into the cavernous sinus and invaded the bone. **d**, **e** Enlarged view of the structures showing the tumor attached to the C4 segment of ICA and M1 segment of MCA and narrowed the lumen of M1

The 3D structures of brain tumor, artery, and bone structure were reconstructed by Mimics software as shown in Fig. 3. The side of the lesion was almost equally distributed (25 right sides, 21 left sides). The tumor volumes ranged from 0.8 to 171.9 cm^3^. Twenty-three (50.0%) tumors were categorized by volume into grade 1, 14 (30.4%) into grade 2, and 9 (19.6%) into grade 3. A total of 39 (84.8%) patients had arterial involvement. Most patients had involvement of the cavernous and/or supraclinoid internal carotid artery (ICA). CS involvement was observed in 23 patients (50.0%). Bone invasion was noted in 10 patients (21.7%). The details of the tumors are presented in Table 2.

**Table 2:** Characteristics of 46 medial sphenoid wing meningiomas

Variables	*N* (%)
Side	
Left	21 (45.7)
Right	25 (54.3)
Tumor volume (cm^3^), mean ± SD (range)	
< 20	9.3 ± 6.0 (0.8–19.5)
20–50	29.9 ± 9.9 (20.2–49.9)
> 50	89.3 ± 39.9 (50.5–171.9)
Total cases of arterial involvement	39 (84.8)
Total cases of multiple arterial involvement	24 (52.1)
Location of arterial involvement	
C4	21 (45.7)
C5	16 (34.8)
M1	21 (45.7)
M2	8 (17.4)
A1	10 (21.7)
A2	1 (2.2)
Cavernous sinus involvement	23 (50.0)
Bone invasion	10 (21.7)

*SD* standard deviation

### Extent of resection

Simpson I resection was achieved in 10 patients (21.7%) and Simpson II resection was achieved in 17 patients (37.0%). Fifteen patients (32.6%) underwent Simpson III resection and 4 patients (8.7%) underwent Simpson IV resections. There was no Simpson V resection in our study (Table 3).

**Table 3:** Surgical outcomes of 46 medial sphenoid wing meningiomas

Variables	*N* (%)
Simpson grade	
I	10 (21.7)
II	17 (37.0)
III	15 (32.6)
IV	4 (8.7)
V	0 (0.0)
Complications postoperative	
CN III palsy	10 (21.7)
CN IV palsy	1 (2.2)
CN V palsy	2 (4.3)
CN VI palsy	1 (2.2)
Hemiparesis	6 (13.0)
Mental disturbance	1 (2.2)
Seizure	1 (2.2)
Visual deterioration	2 (4.3)
Surgical mortality	1 (2.2)
Postoperative evaluation	
0	7 (15.2)
1	27 (58.7)
2	10 (21.7)
3	2 (4.3)

CN, cranial nerve

### Postoperative morbidity and follow-up

Neurosurgical complications occurred in 22 of these 46 patients (47.8%) immediately post-operation. The third nerve palsy was the most frequent complication (10 cases, 21.7%). In most cases (75.0%) of postoperative nerve palsy, the symptoms were temporary. Postoperative hemiparesis occurred in 6 cases (13.0%). In 4 of the 6 patients, hemiparesis improved on long-term follow-up. Details of the complications are listed in Table 3.

Karnofsky Performance Scale (KPS) score has been the gold standard over the years for comprehensive assessment of a patient’s condition. However, it is not applicable to our patients due to the rough classification of each score and cranial palsy was the only neurological deficit in most of our patients post-operation. To evaluate the outcome of surgical treatment, we assigned patients to a 4-category postoperative evaluation system used by Honig et al. [11]. When compared with their preoperative status, 27 patients (58.7%) had fair outcome with unchanged symptoms, whereas 7 patients (15.2%) had worse outcomes with deterioration of symptoms such as visual deterioration, permanent cranial nerve palsy, and hemiparesis. Ten patients (21.7%) had good outcomes with improvement of symptoms such as visual improvement, disappearance of headache or dizziness, and disappearance of seizure. Two patients (4.3%) showed an excellent outcome with complete regression of preoperative tumor-related symptoms. During the long-term follow up, two patients had hydrocephalus post-operation and one of them underwent ventricle-peritoneal shunt for hydrocephalus 7 months post-operation. One patient, aged 69, died of serious pulmonary infection 20 days after operation.

### Evaluation of the proposed scoring system

**Fig. 4:**
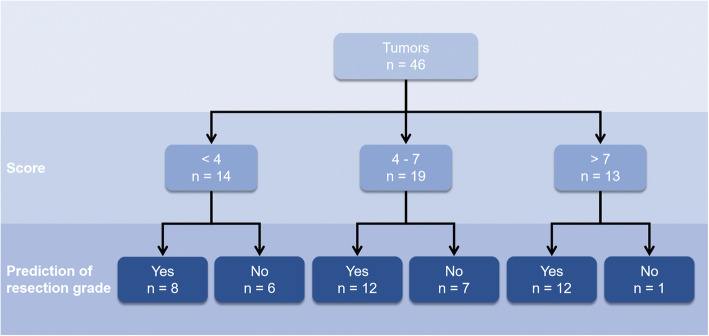
Flow diagram of resection grade predicted by our scoring system

As presented in Table 4, univariate analysis showed that all the variables were significantly associated with Simpson grade of resection (*P* < 0.05). Multivariate analysis showed that only arterial involvement (OR, 2.474; 95% CI, 1.271–4.817) and CS involvement (OR, 13.095; 95% CI, 2.057–83.383) were significantly associated with Simpson grade of resection. Spearman’s analysis showed that both arterial involvement (*R*_s_ = 0.664, *P* < 0.001) and CS involvement (*R*_s_ = 0.711, *P* < 0.001) had strong positive monotonic correlation with resection grade. The tumor volume (*R*_s_ = 0.434, *P* = 0.003) and bone invasion (*R*_s_ = 0.330, *P* = 0.025) also had positive monotonic correlation with resection grade. Considering all these variables’ clinical significance and the correlation among themselves, we included tumor volume, artery encasement, CS involvement, and bone invasion into our scoring system. The details of the scoring system are described in Table 5. The score ranged from 1 to 10. Strong positive monotonic correlation existed between the score and resection grade (*R*_s_ = 0.772, *P* < 0.001). As the score increased, the grade of resection also increased. The mean score of our patients was 5.3 ± 2.8. Discriminant analysis showed that our scoring system had good predictive capacity with an accuracy of 69.60%. A score < 4 often predicted a Simpson I resection, a score between 4 and 7 often predicted a Simpson II resection, while a score > 7 showed possible correlation with a Simpson III or IV resection (Fig. 4). We also found negative monotonic correlation between the score and postoperative evaluation with Spearman’s analysis (*R*_s_ = − 0.433, *P* = 0.003). A score < 4 often indicated a good or excellent outcome. As the score increased, the postoperative evaluation became worse, which might help the surgeon in counseling the patient about potential morbidity.

**Table 4:** Analysis for parameters of resection grade

Parameters	*N* (%)	*P* (univariate analysis)	*P* (logistic regression)	Adjusted OR (95% CI)	*P* (Spearman’s analysis)	Spearman’s correlation coefficient
Volume		0.014	0.889	1.068 (0.425–2.681)	0.003	0.434
1	23 (50.0)					
2	14 (30.4)					
3	9 (19.6)					
Arterial involvement		< 0.001	0.008	2.474 (1.271–4.817)	< 0.001	0.664
0	7 (15.2)					
1	6 (13.0)					
2	8 (17.4)					
3	11 (23.9)					
4	14 (30.4)					
CS involvement		< 0.001	0.006	13.095 (2.057–83.383)	< 0.001	0.711
No	23 (50.0)					
Yes	23 (50.0)					
Bone invasion		0.027	0.122	3.341 (0.724–15.422)	0.025	0.330
No	36 (78.3)					
Yes	10 (21.7)					
Score		< 0.001			< 0.001	0.772
1	5 (10.9)					
2	6 (13.0)					
3	3 (6.5)					
4	6 (13.0)					
5	3 (6.5)					
6	3 (6.5)					
7	7 (15.2)					
8	6 (13.0)					
9	5 (10.9)					
10	2 (4.3)					

Kruskal-Wallis test for ordinal variables and Pearson’s chi-square test for nominal variables to compare between four different resection grade groups. Logistic regression analyses for multivariate analysis. The adjusted OR was obtained by logistic regression. The correlation between the variables and resection grade was detected by Spearman’s correlation analysis.

*OR* odds ratios, *CI* confidence interval

**Table 5:** Details of the scoring system

	Score
Volume	
1	1
2	2
3	3
Arterial involvement	
0	0
1	1
2	2
3	3
4	4
Cavernous sinus involvement	
No	0
Yes	2
Bone invasion	
No	0
Yes	1

Volume: 1, volume < 20 cm^3^; 2, volume between 20 and 50 cm^3^; 3, volume > 50 cm^3^

Arterial involvement: involvement of any artery was given one point, and an additional point was given for each of the following features: completely encircling (360°) the artery, involving more than one artery, and narrowing the lumen of the artery

Cavernous sinus involvement: 0, tumor without cavernous sinus involvement; 2, tumor with cavernous sinus involvement

Bone invasion: 0, tumor without bone invasion; 1, tumor with bone invasion

## Discussion

Medial sphenoid wing meningiomas can invade the cavernous sinus, encircle the vessels, affect nerves, and even invade the bone. These nearby critical anatomic structures can limit the extent of resection, which may result in higher recurrence rates [4, 5, 13, 16]. Therefore, surgical management of mSWM is challenging. In this study, our scoring system, based on preoperative 3D multimodality fusion imaging data of tumor volume, artery encasement, CS involvement, and bone invasion, was able to assess the feature of every tumor intuitively and may provide a preoperative evaluation to predict the extent of resection and help to maximize the safe removal of the tumors and minimize dysfunction.

### Classification of medial sphenoid wing meningiomas

In 1938, Cushing et al. first divided the sphenoid wing meningiomas in detail as “globoid” and “en plaque” tumors. Globoid tumors were further categorized into 3 groups based on their location of origin along the sphenoid wing: (1) deep inner or medial, (2) middle, and (3) lateral. Al-Mefty based his classification of anterior clinoidal meningiomas on the origin of the tumor and whether arachnoid membrane is present [2]. However, the presence of arachnoid membrane cannot be clearly observed preoperatively, thereby limiting its application to our scoring system. Hirsh et al. categorized meningiomas involving CS into three groups based on their relationship to the cavernous carotid to predict the difficulty of resection [10]. Nakamura et al. divided mSWM into 2 groups based on the presence or absence of CS invasion to provide clinical data concerning the visual outcome and recurrence rate [16]. Behari et al. proposed a scoring system for predicting the extent of surgical resection in giant mSWM [3]. However, their study only included 20 patients with giant mSWM (≥ 5 cm in maximum dimension), which may lead to biased results. Moreover, they did not show any statistical analyses of their scoring system. McCracken et al. developed a scoring system for evaluating the degree of encasement of arteries surrounded by the SWM on MRI to predict postoperative ischemic complications [14]. Grade of tumor encasement was determined by the circumferential involvement in details. Recently, Guduk et al. proposed a new scoring system, which included the largest tumor diameter, proximal arterial encasement, distal arterial encasement, and bone invasion pattern, to predict the extent of resection based on preoperative MRI or CT findings [9]. The study included all groups of SWM such as spheno-orbital, medial, middle, and lateral meningiomas. However, they did not perform any evaluation of their scoring system.

None of the studies to date could provide a reliable and overall prediction of surgical resection grade of mSWM based on the preoperative radiology images. The evolution of 3D multimodality fusion imaging has made more accurate guidance for neurosurgery possible. It can clearly reveal the anatomic relationship of the tumor and its surrounding structures and assist in the selection of operative approach and tumor resection. Most of the feeding arteries, perforating arteries, and veins were encased or displaced by the deep-seated meningiomas, which may affect interpretation in 2D images [21]. Our scoring system, based on preoperative 3D multimodality fusion imaging, may provide a more feasible method to predict the resection grade of mSWM and avoid some of the surgical complications. It is also the first study to evaluate mSWM with 3D multimodality fusion imaging.

### Parameters of our scoring system

*Tumor volume* is significantly associated with the extent of resection in our study. Here, we took the meningiomas’ volume calculated by Mimics software rather than the maximum dimension into consideration because most of the meningiomas we analyzed had irregular shapes. Furthermore, there was no significant relationship between the maximum dimension and Simpson excision grade in our cases (*P* = 0.36). Larger meningiomas often had the propensity to invade critical regions and increase the difficulty and risk of surgery [3, 8, 13]. So it is more accurate to analyze the meningiomas’ volume to assess the difficulty encountered during surgery. *Cavernous sinus involvement* is one of the main factors that increase the difficulty of total resection of medially located tumors. As its location is very close to the cranial nerves, the rate of postoperative cranial nerve palsies is consequently higher [24]. The surgical management of tumors involving the CS remains controversial [1, 4, 6, 12, 26]. A more aggressive approach will increase surgical morbidity. But the extent of removal of cavernous sinus meningiomas is inversely related to the rate of recurrence on the other side [4, 16]. In our 23 cases with meningiomas invading into the CS, only 35% of them achieved Simpson II resection, with 88% of the Simpson II resection cases coming out with worse or unchanged symptoms. *Arterial involvement* has a significant effect on the resectability of mSWM. As vessel encasement by tumor increases, the risk of vascular injury will also increase, which may cause higher morbidity and even mortality [14]. When these meningiomas lack the arachnoid plane between the tumor and cerebral vessels, resection becomes more difficult because they can invade the arterial wall, thereby increasing the risk of vascular injury and the difficulty of total removal [2, 20]. In our study, a new classification was developed to include all the possibility of arterial involvement. *Bone invasion* has been found to have an impact on the extent of resection in many studies [3, 9, 22]. The tumor invasion of bone structures such as the superior orbital fissure, optic canal, and orbit is associated with higher risk of morbidity [9] and recurrence [3, 15, 18, 19]. We also found significant difference between bone invasion and resection grade.

All the factors mentioned above had significant association with resection according to our statistical analyses and the surgical experience of our neurosurgeons, especially the arterial encasement and CS involvement. Our scoring system has proved to be effective and convenient to predict the resection grade before surgery. It is the first scoring system for mSWM based on the preoperative 3D multimodality fusion imaging. For patients with higher score, we need to pay more attention to the anatomic relationship between tumor and its surrounding structures. Also, we need to communicate with the patients preoperatively on the higher risk of poor resection grade and poor outcomes. We believe that our scoring system can not only show the advantages to predict the resection grade preoperatively, but also be useful for neurological function protection and reducing postoperative complications.

### Limitation

The tumors’ invasion of venous sinuses increases the difficulty of achieving total excision because it affects the venous drainage and causes the swelling of the brain, which may affect temporal lobe manipulation and thus limit the exposure of tumors. As few of our patients did preoperative magnetic resonance venogram (MRV), we failed to analyze the relationship between venous involvement and resection grade and incorporate the invasion of veins into our score system. The resectability will also depend upon the experience and the aggressiveness of the surgeon. The small sample size presents another major limitation of our study. These factors may influence the resection grade, leading to a bias of our scoring system.

## Conclusions

In this study, we described a reliable preoperative scoring system that enables surgeons to predict extent of resection and postoperative outcomes for mSWM based on 3D multimodality fusion imaging. It may aid neurosurgeons in preoperative planning for mSWM and counseling the patient about potential morbidity.

## Data Availability

The datasets generated during and/or analyzed during the current study are available in the figshare repository. Doi: 10.6084/m9.figshare.11932167

## Abbreviations

CS: Cavernous sinus
CI: Confidence interval
CT: Computed tomography
CTA: Computed tomography angiography
DICOM: Digital Imaging and Communications in Medicine
MRA: Magnetic resonance angiography
MRI: Magnetic resonance imaging
MRV: Magnetic resonance venogram
mSWM: Medial sphenoid wing meningiomas
OR: Odds ratios
